# Lineage-specific targets of positive selection in three leaf beetles correspond with defence capacity against their shared parasitoid wasp

**DOI:** 10.1038/s41437-025-00794-6

**Published:** 2025-09-08

**Authors:** Xuyue Yang, Kalle Tunström, Tanja Slotte, Christopher W. Wheat, Peter A. Hambäck

**Affiliations:** 1 Department of Ecology, Environment and Plant Sciences, Stockholm University, Stockholm, 10691 Sweden; 2 Department of Zoology, Stockholm University, Stockholm, 10691 Sweden; 3 Present Address: Department of Biology, Lund University, Lund, 22362 Sweden

**Keywords:** Evolutionary genetics, Population genetics

## Abstract

Parasitoid wasps are major causes of mortality of many species, making host immune defences a common target of adaptive evolution, though such targets outside model species are poorly understood. In this study, we used two tests of positive selection to compare across three closely related *Galerucella* leaf beetles that show substantial differences in their phenotypic response to the shared parasitoid wasp *Asecodes parviclava*, their main natural enemy. Using a codon-based test, which detects excess amino acid fixations per locus along each species’ lineage, we found more evidence of positive selection on parasitoid-relevant immune genes in the species with the strongest immunocompetence (*G. pusilla*) compared with the species having weaker immunocompetence (*G. tenella* and *G. calmariensis*). Moreover, genes coding for the early phases in the immune response cascade were predominantly among the positively selected immune genes, providing targets for future functional genomic study to pin-point connections between genotypic and phenotypic differences in defences towards a parasitoid wasp. In contrast, genome-wide analyses of the haplotype frequency spectrum, which quantify selection over recent evolutionary time scales, revealed similar signatures of positive selection on immune genes across species. These results advance the field of host-parasitoid dynamics by providing novel insights into the tempo and mode of insect host evolutionary dynamics, and offering a framework for making genotype to phenotype connections for immunocompetence phenotypes.

## Introduction

Parasitoid attacks invariably lead to the death of either the host or the parasitoid, and genes affecting the host’s ability to defend itself against the intruder should therefore evolve rapidly (Kraaijeveld et al. 1998). In insects attacked by endoparasitoid wasps, host survival depends on an immunological reaction that encapsulates the parasitoid egg, thus terminating its development (Carton et al. 2008). Consequently, a major task in evolutionary studies of host-parasitoid systems is to identify the genes underlying phenotypic differences in immunocompetence (Wertheim 2015). Research on model organisms such as *Drosophila melanogaster* and various non-model organisms has identified key pathways involved in this immunological process (Carton et al. 2008). While we know that host species or genotypes may differ greatly in their defence capacity (Carton et al. 2008; Fors et al. 2014; Wertheim 2022), there is limited information outside the *Drosophila* system regarding the role of canonical immune genes as determinants of defence phenotypes.

**Fig. 1:**
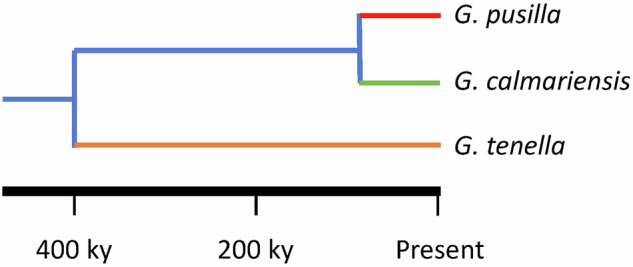
A time-calibrated phylogeny of the three study species and their immune performance against parasitoids (redrawn from Hambäck et al. 2013). Immune performance of each species depicted upon their lineage (red (strongest) > orange > green (weakest); blue not inferred) (cf. Fors et al. 2014).

In our previous work, we have observed large phenotypic differences between three closely related leaf beetle species (Chrysomelidae: *Galerucella* spp.) in defence capacity when attacked by the same parasitoid wasp species (*Asecodes parviclava*, Eulophidae). Whereas *G. pusilla* exhibits a high capacity to encapsulate wasp eggs, this capacity is much lower in *G. tenella* and almost absent in *G. calmariensis* (Fors et al. 2014). These defence phenotypes have been linked to differences in the induction of those immune cells involved in the encapsulation of wasp eggs (Fors et al. 2014) and in the expression of immune-related genes following parasitoid attack (Yang et al. 2020), suggesting a genetic basis for the observed differences in immune performance. These phenotypic differences appear to have evolved since the species diverging approximately 400 ky and 77 ky ago (Fig. 1, Hambäck et al. 2013), presumably in response to the same wasp species, as they lack other specialised enemies.

Here, we use this system, as an opportunity outside of *Drosophila*, to gain broader insights into the genetic basis of defence phenotypes against parasitoids, and to examine the rate of genetic changes between the three *Galerucella* species. Since enemies often vary in their virulence traits, the evolution of host defences also likely varies among species. In *Drosophila*, different parasitoid enemies have been shown to target distinct parts of the immune response pathway (Mortimer 2013), and *Drosophila* species themselves differ in their defence arsenal (Wertheim 2022). In *Galerucella*, key genes underlying major immune pathways in *Drosophila* are present (Yang et al. 2020), providing a useful template for exploring genomic differences that underlie observed difference in defence phenotypes. Previous gene expression analyses indicate that a large number of genes were differentially expressed after parasitoid egg laying in *G. pusilla*, while none were differentially expressed in *G. calmariensis* (Yang et al. 2020). This difference suggests that phenotypic differences are primarily driven by differences in genes coding for the initial phases of the immune response.

Potential candidate genes under selection are likely found among those coding for processes in the cellular immune systems, as this part plays a major role when encapsulating parasitoid wasp eggs (Carton et al. 2008). The other component of the insect immune system, the humoral system, is primarily active against pathogens (Bulet et al. 1999; Gillespie et al. 1997). The common pattern when a parasitoid egg has been injected into the host is that a range of hemocytes are recruited to form a capsule around the egg, after which melanin seals the capsule. This sealing leads to asphyxiation and death of the parasitoid egg. Previous studies suggest that the immune system can evolve rapidly and genes involved in the recognition phase in particular have been found to evolve faster than other genes (Sackton et al. 2007; Schlenke and Begun 2003; Waterhouse et al. 2007). However, most studies to date on selection dynamics have focused on responses to pathogens and the humoral system, with fewer studies addressing parasitoid-specific immune genes. These previous studies have mainly found evidence of positive selection on immune-related genes but also balancing or purifying selection among genes coding for antimicrobial peptides (Heger and Ponting 2007; Unckless et al. 2016; Unckless and Lazzaro 2016; Waterhouse et al. 2007).

In this study, we quantified positive selection on known immune related genes coding for the different functions in the immune pathways. Specifically, we tested the hypothesis that immune related genes would be overrepresented among positively selected genes in the species with the strongest immunocompetence against wasp attack (*G. pusilla*) compared to species with lower immunocompetence (Fig. 1). In addition, based on previous studies, we hypothesised that species differences in positive selection would primarily be observed in genes coding for the early phases in the immune response, such as recognition and signalling. These hypotheses rests upon several complex issues. First, host-parasitoid coevolution is expected to be dynamic, with potentially rapid turnover in the alleles mediating species interactions (e.g., Huang et al. 2021; Sackton et al. 2007). However, it is unclear whether the divergence times of our most derived species provided sufficient time for allelic turnover. Second, our ability to link signatures of positive selection with immune phenotypes depends on assumptions of functional homology based on sequence homology, whereby functional genomics insights from *Drosophila* can inform population genomics results from beetles. Third, molecular tests of selection vary dramatically in their power to detect deviations from neutral expectations, differing in the evolutionary time scales and genomic regions they are able to assess.

To test our hypotheses, we employed two molecular tests (HDMKPRF and LASSI Plus) to detect selection dynamics using population genomic data from three beetle species. Both methods rely on population-level genetic variation but differ primarily in their genomic focus (coding regions vs. genome-wide), and the timescales over which they have the most statistical power. HDMKPRF (high-dimension McDonald-Kreitman Poisson random field method, Zhao et al. 2019) is an extension of the classical MK-test (McDonald and Kreitman 1991) and the MKPRF method developed by Sawyer and Hartl (1992), improving the inference of directionality and the relative strength of selection along lineages unique to each species. LASSI Plus (Harris and DeGiorgio 2020), on the other hand, relies on the site frequency spectrum (SFS), and thus provides insights into more recent selection events. Moreover, HDMKPRF is limited to coding regions, while LASSI Plus detects selection across the entire genome, including the regulatory regions flanking genes. Finally, LASSI Plus estimates haplotype frequency spectrum statistics within sliding windows of population genomic data to detect soft and hard sweeps, with stronger power for the latter category due to the more pronounced signature they leave in the SFS (Harris et al. 2018).

## Material and methods

### Study species

The three *Galerucella* species (Coleoptera: Chrysomelidae) are closely related, with recent divergence times: *G. pusilla* and *G. calmariensis* diverged around 77,000 years ago while *G. tenella* diverged around 400,000 years ago (Fig. 1, Hambäck et al. 2013). *G. pusilla* and *G. calmariensis* are monophagous on *Lythrum salicaria*, whereas *G. tenella* is oligophagous with the primary host *Filipendula ulmaria*. The three beetle species have similar life cycles. Adults appear in the area in May and begin laying eggs on leaves or stems of their host plants. It takes a few weeks for the eggs to hatch, 2–3 weeks for the larvae to pupate, and another 2–3 weeks for the adults to emerge from the pupae. Adults then overwinter until next May. The geographic distribution in Sweden differs between species: *G. pusilla* occurs in the south up to central Sweden (62°N, 17°E) whereas *G. calmariensis* and *G. tenella* occur both in the south and north, along the entire Baltic seashore (Supporting Information, Fig. S1). Within regions, the species commonly co-occur at moderate to high densities, though variable between sites.

The three species share a common endoparasitoid wasp enemy *Asecodes parviclava* (Hymenoptera: Eulophidae), which lays one or more eggs in the beetle larvae (Stenberg and Hambäck 2010). No other parasitoid species attack these beetles in the region. When successfully parasitized, the wasp eggs hatch, and the wasp larvae turn the beetle larvae to a black mummy containing the wasp pupae. However, if the beetles manage to defend themselves, their immune system encapsulates and kills the wasp eggs, allowing the host larvae to continue growing and developing (Fors et al. 2014). Previous studies show that the beetle species differ in their capacity to mount an effective defence against parasitoid attack. While *G. pusilla* exhibits a strong ability to encapsulate wasp eggs, encapsulation is rarely observed in *G. calmariensis* and occurs at an intermediate frequency in *G. tenella* (Fors et al. 2016; Fors et al. 2014). These differences also correspond to selection by wasp females in response to larval odour cues (Fors et al. 2018). The parasitism rates vary between sites and most notably is higher in the northernmost sites, often >50%, compared to southernmost sites, often <10%.

We collected 45 adult individuals, 15 samples from each *Galerucella* species, during May and June 2019 from the following sites: three *G. calmariensis* populations: Norrfjärden (62°3′28″N, 17°26′18″E), Våtnäs (61°32′93″N, 17°12′77″E) and Hölick (61°37′22″N, 17°27′18″E); three *G. pusilla* populations: Rastsjön (60°6′36″N, 17°53′97″E), Lörudden (62°14′14″N, 17°39′12″E) and Haversjön (59°2′31″N, 17°9′49″E); three *G. tenella* populations: Umeå-1 (63°46′72″N, 20°36′00″E), Umeå-2 (63°46′36″N, 20°37′48″E) and Umeå-3 (63°47′18″N, 20°35′89″E). For each population, five individuals were sampled.

### DNA extraction and sequencing

All individual samples were snap-frozen in liquid nitrogen and stored at −80° before DNA extraction. Genomic DNA were extracted from whole adult bodies using KingFisher™ Cell and Tissue DNA Kit following the “DNA Extraction from Single Insects” sample preparation protocol. After extraction, DNA concentrations were measured with a Qubit 3.0 Fluorometer using the dsDNA HS Assay Kit (Thermo Fisher Scientific) and a Nanodrop 8000 to ensure an absorbance ratio at 260/280 between 1.7 and 2. We estimated DNA fragmentation using agarose gel electrophoresis stained with 2% GelRed and only retained samples with minimal degradation. Library preparation was performed with the Illumina TruSeq DNA PCR-free library preparation kit, followed by paired-end 2×150-bp sequencing on a NovaSeq6000 platform at SciLifeLab, Sweden. Library preparation failed for one *G. calmariensis* sample from Våtnäs and this sample was excluded from downstream analysis. The total number of samples with whole-genome resequencing data was thus 44 and we generated 1.6 billion reads of sequence data ( > Q30) in total (out of 1.8 billion reads), corresponding to an average of 34.8 million reads per sample.

### Population mapping and statistics

We assessed the quality of the resequencing data using FastQC v0.11.55 before and after filtering, retaining only reads ≥50 bp with a quality score >30 in both read start and end. All sequence reads were mapped against the *Galerucella calmariensis* reference genome, which was the least fragmented genome (Yang et al. 2021), using NextGenMap version 0.4.12 (Sedlazeck et al. 2013). The reference genome had an assembly size of 588 Mbp, containing 39,255 scaffolds and 40,031 predicted proteins with 91.3% and 85.1% complete orthologs in the genome and proteome, respectively, compared with the endopterygota_odb10 database (Simão et al. 2015) (For further info on the reference genome assembly see Yang et al. 2021). Mapping rates were consistent between samples (85% to 95%). We filtered the resulting bam files with Samtools v1.3.1 (Li et al. 2009) to retain alignments with mapping quality>20 (-q 20).

We next called SNPs across all samples using FREEBAYES v0.9.21 (Garrison and Marth 2012). For SNP filtering of all sites, we only kept bi-allelic sites with a minimum read depth of 5X, a quality score >30 and a maximum proportion of missing data of 20%. Genetic diversity (nucleotide polymorphism, π) was estimated for each species using pixy (Korunes and Samuk 2021). Independent of these steps, we performed a PCA analysis to assess population genetic structure across populations within each species. For this purpose, we first conducted LD-based pruning of our high quality SNPs (--indep-pairwise 50 10 0.2), followed by a principal component analysis (PCA) using Plink v1.9 (Purcell et al. 2007) across all the samples and for each species separately (Figures S2 and S3).

### Testing for positive selection

To detect selection, we employed two molecular tests that rely on population level genetic variation but primarily differ in their genomic focus (coding regions vs. genome-wide), and the time scale over which they have the most statistical power. For coding regions, we used HDMKPRF (Zhao et al. 2019), an extension of MKPRF to analyse selection across multiple species (Bustamante et al. 2001; Sawyer and Hartl 1992). A comprehensive justification for using HDMKPRF to detect genes under selection can be found in Okamura et al. (2022); however, a key advantage is its higher power for detecting weak and moderate selection. Compared to the classical MK-test (McDonald and Kreitman 1991), the greater power of both MKPRF and HDMKPRF arises from adopting a Poisson random-field framework (Sawyer and Hartl 1992), where per gene selection intensities are estimated using a Bayesian approach that combines information across multiple loci and derive posterior distributions. The concept of selection intensities is based on Bustamante et al. (2001) and is akin to a neutrality index measure (Hahn 2019). HDMKPRF improves the MKPRF-test by simultaneously analysing polymorphism and divergence across multiple species, allowing the test to determine in which lineage selection occurred (Zhao et al. 2019). Additionally, HDMKPRF estimates population genetic parameters for each species, such as effective population sizes and mutation rates, and uses these to account for lineage specific differences when estimating selection intensity per locus (for details see Zhao et al. 2019).

To detect positive selection outside the coding regions of genes, we used a maximum likelihood analysis of the haplotype frequency spectrum across the genome to identify putative targets of positive selection via signatures of both soft and hard sweeps. For this, we employed LASSI Plus (Harris and DeGiorgio 2020) and the saltiLASSI statistic (DeGiorgio and Szpiech 2022). This approach is capable of using unphased sequencing data to infer haplotypes and identify genomic regions within population samples that exhibit greater than expected changes in their haplotype allele frequencies, given the background genomic patterns assumed to represent neutrality. This method can estimate both the likelihood of a given haplotype sweeping and the inferred width and number of haplotypes sweeping within a given species. LASSI Plus estimates haplotype frequency spectrum statistics in sliding windows of population genomic data to detect soft and hard sweeps, with stronger power for the latter category due to the more dramatic signature left in the SFS (Harris et al. 2018).

### Codon based test of selection - HDMKPRF

Multiple consensus sequences of coding sequences (CDS) for all samples were extracted using bam2consensus function from BamBam v1.4 (Borowiec 2016), allowing a minimum read coverage per site of 4X. BamBam uses individual bam files, mapped to the *G. calmariensis* draft *de-novo* genome, and extracts consensus sequences for each CDS region based upon the genome annotation. We then assessed summary statistics of the consensus sequences using AMAS v1.0 (Borowiec 2016). Only CDS regions longer than 300 bp and with a low proportion of missing values (<10%) were retained for downstream analysis (*N* = 11,368).

To limit our analysis to orthologous loci among the three species, we assessed orthology between the *G. calmariensis* protein sets and those of three other Coleoptera species (Asian long-horned beetle [*Anoplophora glabripennis*], red flour beetle [*Tribolium castaneum*] and mountain pine beetle [*Dendroctonus ponderosae*]) using OrthoVenn2 (Xu et al. 2019) with default settings. A total of 4591 single-copy-orthologs (SCO) were identified in *G. calmariensis*. These SCOs were cross-references from the previously identified high-quality consensus sequences, resulting in 4154 SCOs for downstream analysis.

Estimates of selection dynamics using synonymous and nonsynonymous polymorphisms and divergence across species can be confounded by rare variants. Such variants could represent weakly deleterious mutations or technical artifacts (e.g., sequencing error). We therefore quantified *α*, the contribution of positive selection to amino acid divergence in genes, for both our full dataset and after singleton removal. The latter increased the estimated proportion of positively selected genes in all three species and reduced indices of negative selection (Table 1). To balance the concerns of quality filtering versus over-filtering through the additional removal of rare variants, we proceeded with selection inferences using the singletons removed dataset. In *G. calmariensis*, we primarily detected positive selection (*α* > 0) and a higher proportion of positively selected genes. Conversely, in *G. tenella*, weak negative selection (*α* < 0) was more prevalent, even after singleton removal, with a higher proportion of negatively selected genes.

**Table 1:** Summary results from the HDMKPRF test among three beetle species.

Species	D_n_	D_s_	P_n_	P_s_	α	Proportion positively/negatively selected sites
Before singleton removal						
*G. calmariensis*	6211	12192	17752	30417	−0.146	12.5%/19.2%
*G. pusilla*	4371	8273	17800	29955	−0.125	8.0%/19.0%
*G. tenella*	3802	7128	20882	33347	−0.174	7.0%/16.5%
After singleton removal						
*G. calmariensis*	6434	12475	9537	18886	0.021	14.4%/16.0%
*G. pusilla*	4503	8444	11469	21630	0.006	10.9%/13.6%
*G. tenella*	3947	7287	13758	23184	−0.096	10.3%/11.3%

The table presents the number of non-synonymous and synonymous polymorphism sites (*P*_*n*_*, P*_*s*_) within species to the ratio of nonsynonymous and synonymous fixed differences (*D*_*n*_*, D*_*s*_) between species summed across all 4154 single copy orthologs. The proportion of nonsynonymous sites fixed by positive selection (*α* = 1-*D*_*s*_*P*_*n*_*/D*_*n*_*P*_*s*_), and the proportion of genes under positive and negative selection estimated by HDMKPRF, for *G. calmariensis*, *G. pusilla* and *G. tenella*. Summary statistics were calculated before and after singleton removal.

The script (Table S1) for performing the HDMKPRF to derive selection intensities was kindly provided by Zhao et al. (2019) The input data for the analysis included all 4154 SCOs and the analysis was implemented by first running 200,000 burn-in steps, followed by estimating posterior parameter distributions from 400,000 steps in a Markov chain Monte Carlo process with a thinning interval of 5, based on the author’s recommendations. A gene was considered to be under positive selection when the 95% posterior credibility interval for the selection intensity was >0, and under negative selection when the interval was <0. Because estimates of positive selection using population resequencing data are usually biased downward by the segregation of slightly deleterious mutations and some singleton errors (Charlesworth and Eyre-Walker 2008), we removed singleton polymorphisms from all gene sets using a custom script (Sattath et al. 2011). We then tested the effect on the power of detecting adaptive evolution by comparing HDMKPRF results for gene sets before and after singleton removal.

### Candidate gene analysis

To specifically analyse immune genes, we first used BLASTP to identify candidate genes from a previous RNA-seq study (Yang et al. 2020) in the *G. calmariensis* proteome. This gene set contains 166 genes suggested to play a role in the immune response to parasitoid wasp attacks in *Drosophila* (Table S2), subdivided into seven functional immune gene categories: recognition (*N* = 17), signalling (*N* = 35), effector (*N* = 21), proteases (*N* = 35), haematopoiesis (*N* = 31), melanisation (*N* = 18), and wound healing (*N* = 9). The threshold used in BLASTP was an E-value ≤ 1 × 10^−6^ and a bitscore>60, which identified a set of 96 immune genes in our genomic dataset. When multiple hits were recovered during the BLAST search, we used the one with the highest bitscore. When incorporating these genes into our SCO gene analysis, only 40 of the immune genes passed the conservative threshold (they are part of the 4154 SCOs gene set). To further focus on the 96 immune genes, we conducted a second HDMKPRF analysis. Given that this second analysis was run on a smaller gene set, HDMKPRF has reduced power. Therefore, we compared the estimated selection intensities for the 40 SCOs between the two analyses and found them to be almost identical, suggesting this set size was sufficient for accurate model parameter estimates.

### Genome-wide test of selection – LASSI Plus

To avoid reference bias in the analysis, we first aligned the short read sequencing data from 15 individuals of each species to their respective reference genomes (Yang et al. 2021) using the bwa-mem2 v.2.0pre2 (Vasimuddin et al. 2019). Variants were then called using bcftools v.1.13-35-ge3ba077 to generate an all-site VCF (Danecek et al. 2021). The resulting VCFs were filtered for low-quality calls (QUAL > 30), read depth (5–50), the exclusion of indels and no more than 2 alternative alleles per site. Inferences of selective sweeps were made using the *salti* statistic in the LASSI Plus software package (k = 10, window size=52 SNPs, step size=12). To identify outlier windows across the genome (i.e, regions likely to have experienced a sweep), we extracted all windows with a *salti* statistic (L) greater than 4 standard deviations above the mean. L is a composite likelihood ratio test statistic indicating that the haplotype frequency spectrum in a given window is distorted relative to genomic background (for statistical code see Table S3).

Finally, for illustrative purposes to compare the distribution and location of selective sweeps between species while avoiding reference bias from aligning samples to a single reference, we scaffolded each species genome against a common chromosome-level assembly from a species in a sister genus, the beetle *Lochmaea crataegi* (NCBI: GCA_947563755.1). Scaffolding was performed using Ragtag v.2.1.0, with default settings, using minimap2 (Alonge et al. 2022), and alignments were filtered to remove any contigs shorter than 50 kb.

### Comparison of results from molecular tests of selection

Comparisons of genome-wide targets of positive selection across species were conducted using two approaches. First, we identified genes within any of the detected sweep regions by intersecting the outlier sweep locations with gene annotations using *bedtools intersect* v2.27.1 (Quinlan and Hall 2010), for each species genome (Yang et al. 2021). Protein sequences of any gene, from start to stop, overlapping any sweep region interval, were then retrieved for each species. We assessed the overlap of the protein sets identified in the three species using Orthovenn2 (Xu et al. 2019), which clusters proteins based on sequence similarity. This approach allows us to identify potential targets of selection shared across the three species, without requiring selection on the exact same gene region, since this analysis accommodates independent members of a gene family being targeted. To determine whether any species had a higher proportion of immune genes among the putative targets of positive selection, we also included the 96 candidate immune genes previously identified in *G. calmariensis* in the Orthovenn2 analysis. Second, we repeated the analysis but extended the candidate gene region by including 5 kb on either side of the gene body (i.e., 5 kb upstream of the start and downstream of the stop) to capture potential signatures of positive selection in regulatory regions. We chose this 10 kb flanking region to account for regulatory evolution based on evidence from other insect groups (Ghavi-Helm et al. 2014; Lewis and Reed 2019).

## Results

### Population-level patterns

PCA of the SNP dataset finds that individuals were tightly clustered by species, with species being distinctly separated (Fig. S2), and minor within-species separation observed between sample sites (Fig. S3). After quality filtering, we retained 7.6 million variable sites out of 126.5 million sites in *G. tenella*, 8.6 million variable sites out of 130.6 million sites in *G. pusilla* and 7.2 million variable sites out of 122.6 million sites in *G. calmariensis*. At the whole genome level, *G. pusilla* populations harboured the highest nucleotide diversity (0.0058), *G. calmariensis* (0.0051) had the lowest, with *G. tenella* (0.0056) having an intermediate nucleotide diversity, estimated as the average number of allelic differences between two haploid genotypes in the same species (Korunes and Samuk 2021).

### Codon based test of selection - HDMKPRF

**Fig. 2:**
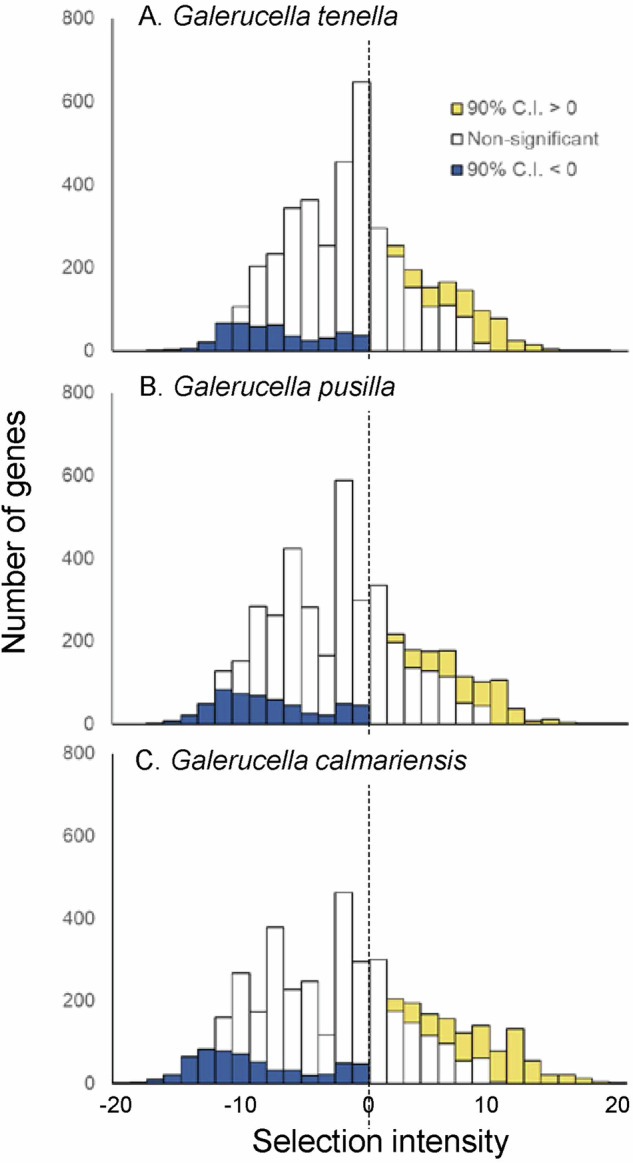
Selection intensities estimated from HDMKPRF. Posterior distributions are shown separately for the three *Galerucella* species (**A** *G. tenella*, **B** *G. pusilla*, **C** *G. calmariensis*) (Blue, 95% CI < 0 = negative selection, Yellow, 95% CI > 0 = positive selection, dotted line = zero selection intensity).

A total of 4154 genes were identified as single-copy loci within the *G. calmariensis* genome, which we used as a reference for estimating polymorphism and divergences across all three species for our codon-based analysis. Posterior mean estimates of the relative effective population sizes (*N*_*e*_) showed a similar pattern as nucleotide diversity, with no dramatic differences among species: *G. pusilla* (N_2_ = 1.045 x N_1_) > *G. tenella* (N_1_) > *G. calmariensis* (N_3_ = 0.934 x N_1_). Out of the 4154 genes, the HDMKPRF method identified a comparable number of genes under selection in all three *Galerucella* species (Fig. 2). In *G. pusilla*, 469 and 562 genes were identified as being under positive and negative selection, respectively. In *G. calmariensis*, these numbers were 665 and 598, respectively, while in *G. tenella* 442 genes were under positive selection and 466 genes under negative selection. Since genes under positive selection are more commonly linked to lineage-specific adaptive traits, we further investigated these loci using gene set enrichment analysis (GSEA, see Supplementary information) and conducted a candidate gene analysis focussed of anti-parasitoid immune genes reported in the literature (Table S2).

### Candidate gene analysis

Our analysis of anti-parasitoid immune genes (96 genes of 166 passed filtering) included 13/17 recognition genes, 21/35 signalling genes, 4/21 effectors, 16/35 protease coding genes, 24/31 haematopoiesis genes, 13/18 melanisation genes and 5/9 wound healing genes. Among these 96 genes, positive selection was detected in seven in *G. pusilla*, which was significantly more than the single gene identified under positive selection in *G. calmariensis* (Fisher’s Exact test: Odds ratio = 0.10, *p* < 0.02), but not significantly greater than the four genes under positive selection in *G. tenella* (Odds ratio = 0.61, *p* > 0.3), when compared to all genes under positive selection in each species. While the four genes under positive selection in *G. tenella* was marginally higher than the one gene in *G. calmariensis*, this difference was not significant (Odds ratio = 0.17, *p* < 0.09). In *G. pusilla*, positively selected genes included those involved in parasitoid recognition (*santa-maria* and *Corin*), Toll and JNK pathways (*grass* and *Tak1*), a protease with serine-type carboxypeptidase activity and genes involved in lamellocyte differentiation (*cher* and *zfh1*) (Table 2, Table S4). The four positively selected immune genes in *G. tenella* included two recognition genes (*Corin* and *PGRP-LE*) and two genes important for haematopoiesis through regulation of lamellocyte differentiation (*cher* and *Cyt-b5*) (Tables 2, S4). The single gene under positive selection in *G. calmariensis* was *Cyp9f2*, which is involved in melanization. Finally, several immune genes were also under negative selection in all three species (Table S4).

**Table 2:** Positively selected immune genes in *Galerucella pusilla* (Gp)*, G. tenella* (Gt) and *G. calmariensis* (Gc).

Species	Recognition	Signalling	Effector	Protease	Haematopoiesis	Melanisation	Wound healing
*Gp*	*santa-maria*	*Tak1*		*CG32483*	*cher*		
	*Corin*	*grass*			*zfh1*		
*Gt*	*Corin*				*cher*		
	*PGRP-LE*				*Cyt-b5*		
*Gc*						*Cyp9f2*	

### Genome-wide test of selection – LASSI Plus

**Fig. 3:**
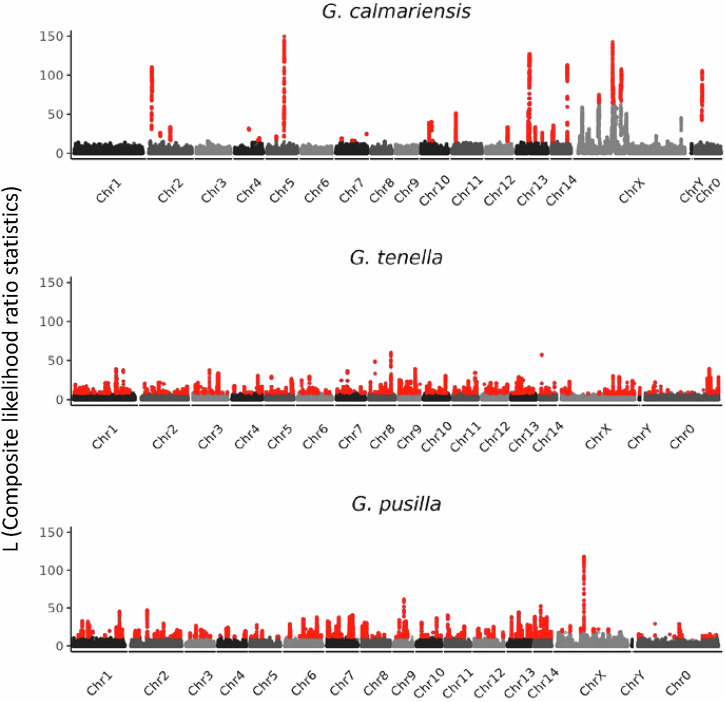
Manhattan plot of haplotype frequency distortion across the genome for three beetle species; contigs were scaffolded based on *Lochmaea crataegi*, a beetle from a sister genus (Chr0 = unscaffolded contigs, ChrX and ChrY = sex chromosomes). Chromosomes are coloured in alternately light and dark grey. Y-axis in each plot is a measure of positive selection, captured by L, a measure of the haplotype frequency distortion. Outlier loci (red dots) were calculated as being more than 4 standard deviations above the mean.

While we were able to detect signatures of selective sweeps in all three species, the patterns were strikingly different between *G. calmariensis* and the other two species. In *G. calmariensis*, the outlier loci were concentrated in a few locations with strong signatures of selection (Fig. 3). In contrast, both *G. tenella* and *G. pusilla* exhibited signatures of selection that were generally weaker, more evenly distributed across their genomes and narrower in size (Fig. 3). To make a direct comparison with the HDMKPRF test, which focuses on the coding region of genes, we identified genes that had a direct overlap of the sweep region with any part of a gene body, from the start to the stop codon. This intersection of outlier haplotypes with annotated genes revealed 192, 115, and 154 genes in *G. calmariensis, G. pusilla* and *G. tenella*, respectively. Since targets of positive selection might act upon the regulatory regions of genes, rather than the coding or intronic regions, we extended our gene targets by 10 kb on either side of each gene. When intersecting these gene bodies and their regulatory regions with the outlier haplotypes, 216, 305, and 430 genes were intersected. The increase in detected outliers of the latter two species, when including likely regulatory regions flanking genes, suggests more overlap with potential regulatory regions in those species. Next, we assessed the extent to which candidate targets of positive selection identified in the genome-wide analysis overlapped with our candidate immune genes. Unlike the results for the HDMKPRF test, these outlier genes (with 10 kb on either side) were roughly equally distributed among the three species (7, 6, and 6 genes in *G. calmariensis, G. pusilla* and *G. tenella*, respectively).

### Comparison of results from molecular tests of selection

The molecular tests of selection employed in this study query different genomic regions (coding vs. whole-genome), detect distinct signatures of deviations from neutrality (accumulation of nonsynonymous substitutions vs. shifts in haplotype frequencies), and assess selection dynamics across different time scales (a species lineage vs. current site frequency spectrum). These differences complicate efforts to directly compare their results. Additionally, comparisons are made more challenging by our efforts to minimise the inherently different sensitivities of each method to dataset construction.

Our codon level test (HDMKPRF) is highly sensitive to gene model accuracy across all species analysed, requiring strict locus orthology when quantifying genetic variation in the coding sequence (CDS) region. In contrast, our haplotype frequency-based test (LASSI Plus) is a within-species analysis that is primarily sensitive to reference bias, which we minimised by mapping sequencing reads from each species to its own genome. For HDMKPRF, strict orthology was achieved by mapping population data from all three species to the most complete genome (*G. calmariensis*), limiting direct comparisons of LASSI Plus to this species. In *G. calmariensis*, 595 genes were identified as having experienced positive selection via HDMKRPF, with only 10 of these genes overlapping with the 216 genes located within haplotype sweep regions identified by LASSI Plus. None of these overlapping genes were immune-related.

Because the tests detect selection at different regions and over different time scales, there are only a limited number of scenarios where they would identify overlapping outlier regions. Departures from neutrality detected by HDMKPRF are expected to result from repeated selective sweeps of favoured nonsynonymous mutations, which LASSI Plus would only detect if such a sweep has recently occurred in the sampled population. Beyond evaluating the exact orthologs identified by both tests, the two approaches revealed distinct patterns of selection dynamics. HDMKPRF consistently found that the relative proportion of positively selected immune genes mirrored the relative immunocompetence of our three species (Fig. 1), whereas LASSI Plus identified similar proportions of positively selected genes across species.

## Discussion

A successful parasitoid attack results in host death, and parasitoids can therefore be expected to exert strong selection on hosts to develop defence mechanisms. In this paper, we examined signatures of positive selection to gain insights into the evolutionary dynamics from a single parasitoid shared on three closely related host species. Importantly, our previous work documents differences in defence phenotypes among these hosts when attacked by the same parasitoid wasp. *Galerucella pusilla* exhibits a very strong immune response, *G. calmariensis* has a very weak immune response whereas *G. tenella* shows an intermediate immune response. In our between-species analysis, which detects strong signatures of positive selection, we found that the ranking of host defence performance aligned with the number of immune genes showing signs of positive selection. However, a within-species analysis that detects only recent selective sweeps revealed no such relationship. While these findings suggest a temporal difference in the selection dynamics among species, many additional factors warrant discussion, including the role of microbial pathogens in the selection dynamics. Below, after placing our findings of positive selection in the context of the broader literature, we address several caveats that should be considered.

### Identification of candidate genes underlying defence phenotypes

The HDMKPRF tests revealed that seven immune genes were under positive selection in *G. pusilla*, the species with the strongest ability to encapsulate wasp eggs, compared with just one immune gene under positive selection in *G. calmariensis*, which lacks the capacity to encapsulate wasp eggs, and four immune genes in *G. tenella*, the species having an intermediate encapsulation capacity. These findings suggest that evolutionary adaptations in host defences are driven not only by changes in regulatory regions but also by modifications in protein-coding regions. Although the identified genes have yet to be functionally characterised in these species, their relevance in mediating defence against parasitoids has been partly confirmed by previous comparative gene expression analyses (Yang et al. 2020). In contrast, LASSI Plus identified an equal number of immune related genes as outliers across the three species. However, it is notable that most of those genes (4/6) identified as outliers in *G. pusilla* by LASSI Plus were previously found to be differentially expressed following wasp attack whereas none of the genes in *G. calmariensis* showed such differential expression.

Although we analysed only a subset of immune genes in the *Galerucella* genome (96 out of 166), both the current study and the previous gene expression analysis suggest that species differences in defence phenotypes are best explained by genes coding for proteins involved in the early phases of the immune process. First, the gene expression analysis showed that multiple genes were upregulated in *G. pusilla* when attacked by *A. parviclava*, whereas almost none were upregulated in *G. calmariensis* (Yang et al. 2020). Second, most of the genes under positive selection in the HDMKPRF analysis encode proteins involved in wasp egg detection and the recruitment of hemocytes to build the capsule, while genes related to melanization or wound healing processes showed less evidence of positive selection. We identified several promising candidate genes potentially involved in recognition or signalling processes, such as *santa-maria*, *Corin*, *PGRP-LE*, *Tak1* and *Grass*. Notably, two of these genes (*Grass* and *PGRP-LE*) were also identified as outliers in the LASSI Plus analysis when including flanking regions. While the specific functions of these five genes in *Galerucella* are unknown, some insights are available from studies on *Drosophila*. For example, both *santa-maria* and *Corin* are thought to encode proteins with scavenger receptor or serine-type endopeptidase activities (Cao and Jiang 2018), with *Corin* containing a transmembrane domain that may function as a receptor to the Toll pathway (Irving et al. 2001). Although the role of *Corin* remains unclear, studies on *Drosophila* show its upregulation following bacterial infections (Irving et al. 2001), though not in response to parasitoid attack (Salazar Jaramillo et al. 2017). Similarly, *PGRP-LE* encodes an intracellular protein that binds to diaminopimelic acid-type peptidoglycans to activate the IMD/Relish pathway (Bosco-Drayon et al. 2012), though its primary function may be anti-bacterial (Libert et al. 2006). *Grass*, meanwhile, has been predicted to be involved in the Toll signalling pathway, which is considered crucial in the immune response against wasp attack in *Drosophila* (Carton et al. 2008; Lemaitre and Hoffmann 2007; Wertheim et al. 2005), while *Tak1* may regulate the switch between the JNK and IMD pathways. Among these genes, *Corin* stands out as particularly interesting, as it was under positive selection in both *G. pusilla* and *G. tenella*, the two species with relatively more efficient encapsulation responses. This pattern suggests that *Corin* may have evolved twice: once in *G. tenella* following its split from the common ancestor of *G. calmariensis* and *G. pusilla*, and again in *G. pusilla* after its divergence from *G. calmariensis*.

While recognition and signalling genes are sufficient to explain differences in defence phenotypes, we also detected positive selection on genes involved in the downstream regulation of hemocytes. Notably, *cher* was under positive selection in both *G. tenella* and *G. pusilla*, and has previously been shown to negatively regulate lamellocyte differentiation (Rus et al. 2006). Lamellocytes are key components in the capsules that kill parasitoid eggs of *Galerucella* and are induced from precursors following attack by *A. parviclava* (Fors et al. 2016; Fors et al. 2014). A similar response was observed in *Drosophila* following parasitoid attack (Kim-Jo et al. 2019). Two other genes involved in lamellocyte regulation also showed evidence for positive selection, *zfh1* in *G. pusilla* and *Cyt-b5* on *G. tenella*. *zfh1* is part of a transcription factor cascade that acts as a switch between plasmatocyte and lamellocyte differentiation (Frandsen et al. 2008), and *Cyt-b5* encodes a conserved hemoprotein required for hemocyte regulation (Kleinhesselink et al. 2011). Both of these genes were also found to be differentially expressed following infection by *A. parviclava* in *G. pusilla* (Yang et al. 2020).

### Caveats and limitations

The tools used in our analysis rely on different evolutionary dynamics to detect positive selection, with diverse processes potentially contributing to the observed differences among tests. First, immune genes are known to exhibit strong variation in gene copy number due to high rates of gene duplication (Sackton et al. 2017), especially for recognition and effector genes, though less so for signalling genes (Lazzaro 2008). Second, our focus was on detecting directional selection, and we did not test for balancing selection, which could underlie red queen dynamics (e.g., Bourgeois et al. 2021). Third, in the LASSI Plus analysis, we included 10 kb flanking regions around coding sequences, but this test would not capture changes in *trans*-regulatory regions, which have been suggested to influence the evolution of *Drosophila* immune systems (Ding et al. 2022).

The current analyses represent a first step in understanding the evolutionary processes driving trait evolution in this system. However, there are several methodological concerns in the HDMKPRF analysis that warrant attention. First, we excluded genes with low coverage or those affected by recent gene birth-death dynamics, limiting our analysis to 4154 orthologous genes. This restriction may have reduced our ability to detect immune-associated genes. While it is unclear if these exclusions biased our conclusions, this smaller, high-confidence gene set – free from recent gene duplications and low-coverage issues – provides a robust basis for inferring selection dynamics and is sufficient for estimating genome-wide patterns. Second, a general challenge in MK tests is their sensitivity to demographic history (Eyre-Walker 2002; McDonald and Kreitman 1991). We approached this problem by using HDMKPRF, which attempts to correct for demography effects during the detection of selection patterns by accounting for changes in effective population size and other population genetic parameters. Third, if deleterious mutations are segregating in the population, the degree of positive selection could be underestimated. To address this, we followed the widely accepted practice of removing deleterious mutations by setting a threshold to filter out minor alleles (Charlesworth and Eyre-Walker 2008). Removing only singleton polymorphisms increased the proportion of negatively selected sites but preserved the relative ranking of α (the proportion of adaptive substitutions) among species. Thus, while our absolute α-estimates are likely underestimated, the relative ranking among species are probably accurate. Nonetheless, differences in effective population size (*N*_*e*_) may also affect α, and the lower *N*_*e*_ for *G. calmariensis* could explain the higher α observed for this species.

In contrast to our predictions and the results from the HDMKPRF test, LASSI Plus failed to detect an enrichment of immune genes between species. Since the LASSI plus analysis is not restricted to genic regions, this discrepancy is not due to our focus on either regulatory or coding regions for sweep detection. A more likely explanation for the differing results is that HDMKPRF is a more conservative test; it incorporates all fixation events since the last common ancestor and requires multiple rounds of selective sweeps to achieve significant departures from neutral expectations. In contrast, LASSI Plus, like nearly all tests relying on outliers in the site frequency spectrum, can only detect positive selection events on a much more recent time horizon, likely reflecting only a small fraction of the time since the last common ancestor. While limited in the time horizon it can assess, LASSI Plus has substantial power to detect strong soft and hard sweeps throughout the genome. In summary, while the HDMKPRF test is narrowly focused on selection acting on amino acid variation, it benefits from a broader time horizon. Conversely, LASSI Plus, despite its ability to detect positive selection across the genome, is limited to events occurring in the relatively recent past.

While these two methods are powerful molecular tests of selection, two limitations reducing power in our analysis are the limited sample sizes and the fragmented reference genomes. Even though these tools are able to account for some demographic effects and for detecting the common variants in each species, deeper sampling may have been needed to recover additional informative sites and to detect rare variants. Despite this problem, we are confident that our analysis reflects species differences due to the uniform analysis across species. The fragmented genome assembly mainly reduced power in LASSI Plus, because this issue affects haplotype based molecular test of selection, likely leading to an underestimation of genomic regions experiencing positive selection. Nevertheless, we believe that this problem had limited effects on the analysis, both because the effect only occurs for cases where we expect rather large haplotype blocks under selection but also due to the high rates of recombination in these species.

## Conclusion

Parasitic wasps and other parasitoids have been extremely successful, with some studies suggesting that their global species number may surpass those of any other arthropod group (Forbes et al. 2018). This high diversity makes host-parasitoid interactions among the most common types of species interaction. It is likely that these interactions have influenced the evolutionary trajectories of both parasitoids and their hosts; however, our understanding of the reciprocal evolutionary process outside the *Drosophila* model system remains limited. In this study, we focussed on the host side of the interaction, taking advantage of the observed phenotypic differences in defence capacity among three closely related leaf beetle species. While the species with the strongest immunocompetence exhibited the most significant evidence of positive selection on immune related genes, this pattern was not consistent across different analytical methods. Tests targeting more recent selection events found no such trend. At this stage, the historical evolutionary dynamics can only be speculated upon. One emerging hypothesis is that phenotypic differentiation resulted from geographic isolation, leading to different coevolutionary trajectories induced by parasitoids in the various host species (cf. Buckling and Hodgson 2007; Buckling and Rainey 2002). The genes identified as likely to have experienced positive selection suggest that selection may act on multiple components within the immune pathways (Kraaijeveld et al. 1998). Consequently, the selection dynamics shaping the immune system of the beetles are likely ancient, with the observed phenotypic and genotypic differences arising during or after the speciation process in a coevolutionary context between hosts and parasitoid wasps. Further understanding of the immune response likely necessitates functional analyses using gene editing through CRISPR-Cas.

## Data archiving

Sequence data generated and analysed during the current study have been deposited at the European Nucleotide Archive (http://www.ebi.ac.uk/ena) under the accession PRJEB56839.

## Supplementary information

Supporting information
