# Supplementary material for: Lineage-specific targets of positive selection in three leaf beetles correspond with defence capacity against their shared parasitoid wasp
Source: Heredity (Edinb). 2025 Sep 8;134(9):567–75. doi: 10.1038/s41437-025-00794-6 (PMC12457636; doi:10.1038/s41437-025-00794-6)
Supplement: Supplementary file 1 — Supporting information [file 41437_2025_794_MOESM1_ESM.docx]

**Supporting information**

Gene set enrichment analysis (GSEA)

The GSEA was implemented with a Gene Ontology (GO) enrichment test of biological process terms of genes under selection using the BIOCONDUCTOR package topGO (Alexa and Rahnefuhrer 2010). The background gene set included the 4,154 genes analysed in HDMKPRF and those genes identified as significantly selected (positively or negatively) were tested for enrichment of biological process terms (FDR<10%) against this background using the parent–child algorithm (Grossmann, et al. 2007). To ensure robust results, we only analysed GO terms with at least five members (node size=5). Prior to analysis, we used EggNOG v5.0 (Huerta-Cepas, et al. 2019) to assign GO terms to the predicted protein sets, restricting the function inferences to the insect-related scale by using Insecta as the taxonomic scope (see supplementary information). Subsequently, enriched GO terms were clustered into representative functional subsets using the REVIGO *Drosophila* database (Supek, et al. 2011).

GSEA found several functions that were enriched in genes under positive selection, some of which were common among the three beetle species and some that differed (Tables S3-S5). First, enriched functions common among the beetle species included functions involved in the formation of adult morphology, such as the imaginal disc pattern formation (forming the adult cuticle and appendage structures), the wing disc pattern formation (forming wing structures) and the dorsal/ventral pattern formation. Second, unique gene categories under positive selection in *G. calmariensis* included those coding for metabolic processes (e.g., processes related to carbohydrate derivatives, oligosaccharides, amino sugars, sulphur compounds and catechol-containing compounds) and those coding for processes in the nervous system (e.g., neuroblast proliferation, neuroblast differentiation, nervous system process) (Table S3). Third, unique gene categories under positive selection in *G. pusilla* included those coding for positive regulation of the innate immune response, including positive regulation of small GTPase mediated signal transduction, and those coding for axoneme assembly (e.g., cilium movement, cilium organization) (Table S5). Finally, unique gene categories under positive selection in *G. tenella* included genes coding for a range of biosynthetic processes (e.g., nucleobase-containing compound biosynthetic processes, heterocycle biosynthetic processes and aromatic compound biosynthetic processes), genes coding for lipid metabolic processes (e.g., sterol metabolism, membrane lipid biosynthesis, cellular lipid metabolism, sphingolipid metabolism and lipoprotein metabolism), but also GO pathways involving the activation of immune response, pigment metabolic process involved in pigmentation, peripheral nervous system development and response to oxidative stress (Table S4).

When comparing the GSEA results for immune related terms, *G. tenella* had a weaker signal (GO:0002253 activation of immune response, -log10Pvalue = 1.43) compared to *G. pusilla* (GO:0045089 positive regulation of innate immune response, -log10Pvalue = 1.95). In order to move beyond these P-value estimates of relative enrichment, and gain more insights into the immune genes that are involved in host response to parasitoid attack, we conducted a candidate gene analysis of anti-parasitoid immune genes from the literature (Table S2).

When examining other gene functions identified as experiencing positive selection, we found that genes coding for imaginal disc pattern formation and wing disc pattern formation were under positive selection in all species. Imaginal discs are epithelial sacs found in insect larva that later develop into cuticular structures (e.g., head, wing, limbs, thorax) of adult insects, and the wing disc is among the largest imaginal discs in insects (Blair 2009). These findings are not surprising as the beetle species are morphologically differentiated, both in size and colour. Other genes varied between species, but those under positive selection in *G. calmariensis* specifically involved genes coding for metabolic functions such as the metabolism of carbohydrate derivatives, oligosaccharides, amino sugars, sulphur compounds and catechol-containing compounds. These sets of positively selected gene functions indicate the importance of energy allocation and dietary transitions during evolution in this species. Moreover, pathways related to the nervous systems were also found to be positively selected in *G. calmariensis*. Positive selection on nervous system-related genes were previously documented in social insects such as bees and ants (Roux, et al. 2014; Woodard, et al. 2011) but have rarely been reported in beetles. These patterns may indicate changes either in the capacity to detect host plants or mates and may thus be involved in the species differentiation. In *G. tenella*, several pathways related to lipid metabolic processes were enriched in positively selected genes. Some of these processes, such as sterol metabolism, may be linked to needs to handle differences in plant chemistry from different host plants (Rosaceae vs. *L. salicaria*). Other lipids, such as sphingolipids, have been suggested to be involved in cell defences and could be interesting to study in relation to wasp attack. Finally, a unique pathway under positive selection in *G. tenella* is involved in the pigmentation metabolic process, which may potentially underlie the different spotting and pigmentation patterns in the species.

**
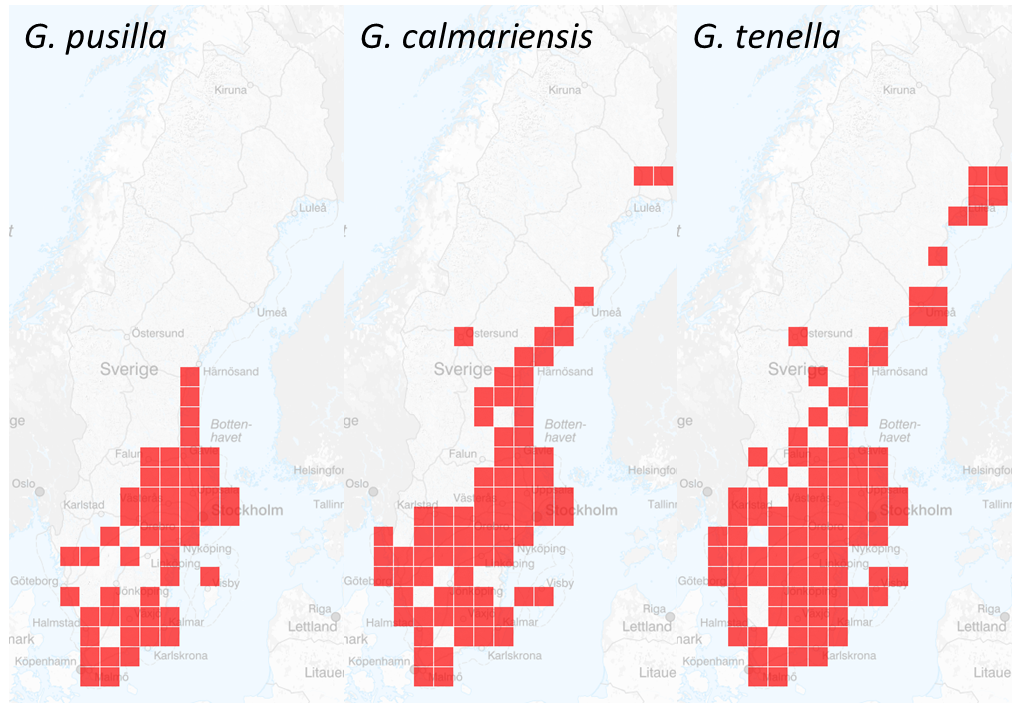
**

Figure S1. Distributions of the three species in Sweden. Maps from https://artportalen.se

**Figure S2.** PCA plot of populations from three *Galerucella* species. Species are represented by colors: red: *G. pusilla* (N = 15); green: *G. tenella* (N = 15); blue: *G. calmariensis* (N = 14).

**
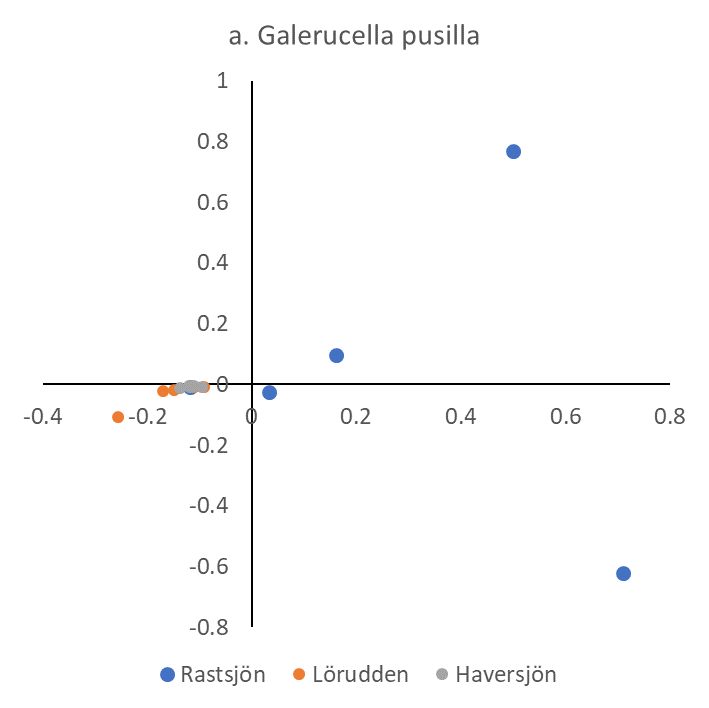
**

**
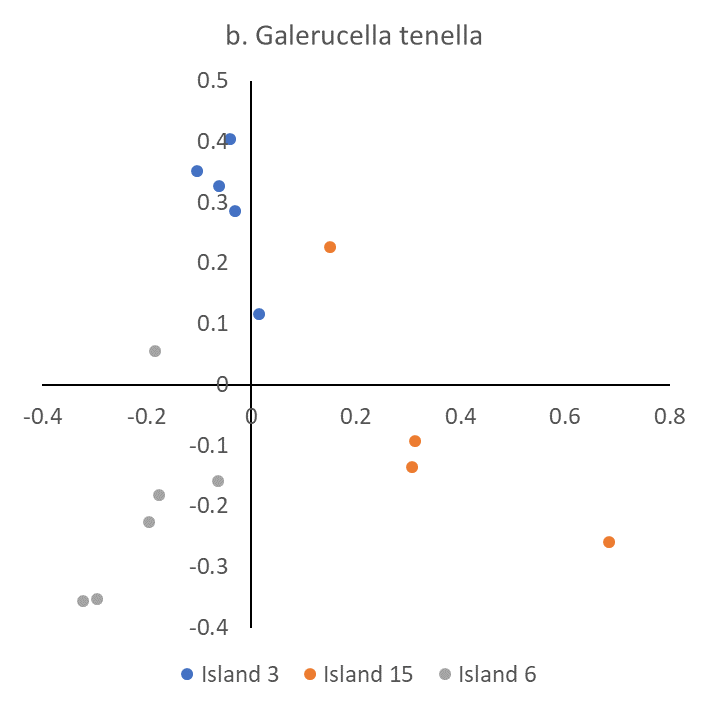
**

**
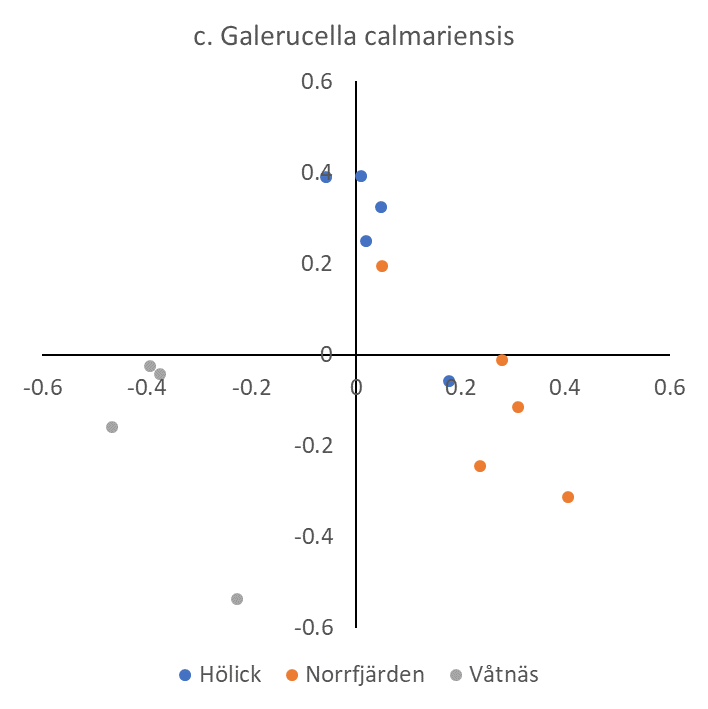
**

**Figure S3.** PCA plot separated by species. Populations within species are separated by colors. Site information - *G. calmariensis*: Norrfjärden (62°3'28"N, 17°26'18"E), Våtnäs (61°32'93"N, 17°12'77"E ) and Hölick (61°37'22"N, 17°27'18"E); *G. pusilla*: Rastsjön (60°6'36"N, 17°53'97"E ), Lörudden (62°14'14"N, 17°39'12"E) and Haversjön (59°2'31"N, 17°9'49"E); *G. tenella*: Umeå-1 (63°46'72"N, 20°36'00"E ), Umeå-2 (63°46'36"N, 20°37'48"E ) and Umeå-3 (63°47'18"N, 20°35'89"E ).

**Table S1.** Code for running HDMKPRF

# -p path of the folder contains fasta files

# -bs (int)number of burn-in steps(default:50000)

# -ts (int)total steps of MCMC(default:100000)

# -si (int)sample interval after burn-in step(default:10)

# -order (string)(optional)path to order file(Non-mandatory), order of phylogeny

# $inputdir contains fasta sequence files of all the orthologs. For each ortholog, reads of tenella comes first and then pusilla then calmariensis, which followed the phylogeny (# The phylogenetic relationships between the three species: ((G. calmariensis, G. pusilla)G. tenella)

#run HDMKPRF

#HDMKPRF_linux -p $inputdir -bs 200000 -ts 400000 -si 5 -o .out

**Tables S2.** List of 166 candidate anti-parasitoid immune genes. Functions were inferred from previous studies in *Drosophila*. Those underlined were retained in the analysis.

| **Gene** | **Gene full name** | **CG** | **Function** |
| --- | --- | --- | --- |
| alphaPS4 | Integrin alphaPS4 subunit | CG16827 | Recognition |
| Corin | Corin | CG2105 | Recognition |
| GNBP3 | Gram-negative bacteria binding protein 3 | CG5008 | Recognition |
| Hml | Hemolectin | CG7002 | Recognition |
| lectin-24A | lectin-24A | CG3410 | Recognition |
| PGRP-LB | Peptidoglycan recognition protein LB | CG14704 | Recognition |
| PGRP-LE | Peptidoglycan recognition protein LE | CG8995 | Recognition |
| PGRP-SA | Peptidoglycan recognition protein SA | CG11709 | Recognition |
| PGRP-SB1 | Peptidoglycan recognition protein SB1 | CG9681 | Recognition |
| PGRP-SD | Peptidoglycan recognition protein SD | CG7496 | Recognition |
| santa-maria | scavenger receptor acting in neural tissue and majority of rhodopsin is absent | CG12789 | Recognition |
| Tep1 | Thioester-containing protein 1 | CG18096 | Recognition |
| Tep3 | Thioester-containing protein 3 | CG7068 | Recognition |
| Tep2 | Thioester-containing protein 2 | CG7052 | Recognition |
| Tep4 | Thioester-containing protein 4 | CG10363 | Recognition |
| CG13422 | CG13422 | CG13422 | Recognition |
| CG2736 | CG2736 | CG2736 | Recognition |
| 18w | 18 wheeler | CG8896 | Signalling |
| bsk | basket | CG5680 | Signalling |
| cactus | cactus | CG5848 | Signalling |
| dl | dorsal | CG6667 | Signalling |
| Dif | Dorsal-related immunity factor | CG6794 | Signalling |
| DmMyD88 | Myd88 | CG2078 | Signalling |
| dome | domeless | CG14226 | Signalling |
| dredd | Death related ced-3/Nedd2-like caspase | CG7486 | Signalling |
| grass | Gram-positive Specific Serine protease | CG5896 | Signalling |
| Hep | hemipterous | CG4353 | Signalling |
| hop | hopscotch | CG1594 | Signalling |
| IKKβ | I-kappaB kinase β | CG4201 | Signalling |
| imd | immune deficiency | CG5576 | Signalling |
| Jra | Jun-related antigen | CG2275 | Signalling |
| kenny | kenny | CG16910 | Signalling |
| nec | necrotic | CG1857 | Signalling |
| Os | unpaired 3 | CG33542 | Signalling |
| pll | pelle | CG5974 | Signalling |
| puc | puckered | CG7850 | Signalling |
| psh | persephone | CG6367 | Signalling |
| Rel | Relish | CG11992 | Signalling |
| SPE | Spatzle-Processing Enzyme | CG16705 | Signalling |
| Socs36E | Suppressor of cytokine signaling at 36E | CG15154 | Signalling |
| spheroide | spheroide | CG9675 | Signalling |
| sphinx1 | sphinx1 | CG32383 | Signalling |
| spirit | Serine Protease Immune Response Integrator | CG2056 | Signalling |
| spz | spatzle | CG6134 | Signalling |
| Stam | Signal transducing adaptor molecule | CG6521 | Signalling |
| Stat92E | Signal-transducer and activator of transcription protein at 92E | CG4257 | Signalling |
| Tak1 | TGF-β activated kinase 1 | CG18492 | Signalling |
| Toll | Toll | CG5490 | Signalling |
| Toll-6 | Toll-6 | CG7250 | Signalling |
| Toll-7 | Toll-7 | CG8595 | Signalling |
| Tollo | Tollo | CG6890 | Signalling |
| tub | tube | CG10520 | Signalling |
| AttA | Attacin-A | CG10146 | Effector |
| AttB | Attacin-B | CG18372 | Effector |
| AttD | Attacin-D | CG7629 | Effector |
| CecC | Cecropin C | CG1373 | Effector |
| Cyp6a17 | Cyp6a17 | CG10241 | Effector |
| Cyp9c1 | Cytochrome P450-9c1 | CG3616 | Effector |
| Cyp12a4 | Cyp12a4 | CG6042 | Effector |
| Cyp12e1 | Cyp12e1 | CG14680 | Effector |
| DHPR | Dihydropteridine reductase | CG4665 | Effector |
| Fmo-2 | Flavin-containing monooxygenase 2 | CG3174 | Effector |
| IM1 | Immune induced molecule 1 | CG18108 | Effector |
| IM2 | Immune induced molecule 2 | CG18106 | Effector |
| IM3 | Immune induced molecule 3 | CG16844 | Effector |
| IM4 | Immune induced molecule 4 | CG15231 | Effector |
| mthl2 | methuselah-like 2 | CG17795 | Effector |
| Mtk | Metchnikowin | CG8175 | Effector |
| Pxn | Peroxidasin | CG12002 | Effector |
| TotA | Turandot A | CG31509 | Effector |
| TotB | Turandot B | CG5609 | Effector |
| TotC | Turandot C | CG31508 | Effector |
| CG15065 | CG15065 | CG15065 | Effector |
| spn28D | Serpin 28Dc | CG7219 | Protease |
| spn4 | Serpin 42Da | CG9453 | Protease |
| Spn88Eb | Serpin 88Eb | CG6687 | Protease |
| Tequila | Tequila | CG4821 | Protease |
| λTry | λTry | CG12350 | Protease |
| CG11313 | CG11313 | CG11313 | Protease |
| CG11912 | CG11912 | CG11912 | Protease |
| CG12951 | CG12951 | CG12951 | Protease |
| CG16713 | CG16713 | CG16713 | Protease |
| CG17278 | CG17278 | CG17278 | Protease |
| CG17475 | CG17475 | CG17475 | Protease |
| CG17572 | CG17572 | CG17572 | Protease |
| CG18563 | CG18563 | CG18563 | Protease |
| CG30090 | CG30090 | CG30090 | Protease |
| CG30371 | CG30371 | CG30371 | Protease |
| CG30414 | CG30414 | CG30414 | Protease |
| CG3117 | CG3117 | CG3117 | Protease |
| CG31269 | CG31269 | CG31269 | Protease |
| CG31780 | CG31780 | CG31780 | Protease |
| CG31827 | CG31827 | CG31827 | Protease |
| CG32374 | CG32374 | CG32374 | Protease |
| CG32376 | CG32376 | CG32376 | Protease |
| CG32483 | CG32483 | CG32483 | Protease |
| CG3344 | CG3344 | CG3344 | Protease |
| CG3505 | CG3505 | CG3505 | Protease |
| CG3916 | CG3916 | CG3916 | Protease |
| CG4053 | CG4053 | CG4053 | Protease |
| CG4653 | CG4653 | CG4653 | Protease |
| CG4793 | CG4793 | CG4793 | Protease |
| CG5246 | CG5246 | CG5246 | Protease |
| CG6041 | CG6041 | CG6041 | Protease |
| CG6639 | CG6639 | CG6639 | Protease |
| CG9240 | CG9240 | CG9240 | Protease |
| CG9673 | CG9673 | CG9673 | Protease |
| CG9733 | CG9733 | CG9733 | Protease |
| Adgf-A | Adenosine deaminase-related growth factor A | CG5992 | Hematopoiesis |
| brm | Brahma | CG5942 | Hematopoiesis |
| cher | Cheerio | CG3937 | Hematopoiesis |
| chn | Charlatan | CG11798 | Hematopoiesis |
| Cyt-b5 | Cytochrome b5 | CG2140 | Hematopoiesis |
| Dom | Domino | CG9696 | Hematopoiesis |
| Dpp | decapentaplegic | CG9885 | Hematopoiesis |
| Dronc | Death regulator Nedd2-like caspase | CG8091 | Hematopoiesis |
| Dsor1 | Downstream of raf1 | CG15793 | Hematopoiesis |
| Egfr | Epidermal growth factor receptor | CG10079 | Hematopoiesis |
| gcm | glial cells missing | CG12245 | Hematopoiesis |
| He | Hemese | CG31770 | Hematopoiesis |
| kn | knot | CG10197 | Hematopoiesis |
| lwr | lesswright | CG3018 | Hematopoiesis |
| lz | lozenge | CG1689 | Hematopoiesis |
| Mxc | multi sex combs | CG12124 | Hematopoiesis |
| Notch | Notch | CG3936 | Hematopoiesis |
| pnt | pointed | CG17077 | Hematopoiesis |
| Pvf2 | PDGF- and VEGF-related factor 2 | CG13780 | Hematopoiesis |
| Pvr | PDGF- and VEGF-receptor related | CG8222 | Hematopoiesis |
| Rac1 | Rac1 | CG2248 | Hematopoiesis |
| raf | Raf oncogene | CG2845 | Hematopoiesis |
| Ser | Serrate | CG6127 | Hematopoiesis |
| sgg | shaggy | CG2621 | Hematopoiesis |
| srp | serpent | CG3992 | Hematopoiesis |
| Su(H) | Supressor of Hairless | CG3497 | Hematopoiesis |
| ush | u-shaped | CG2762 | Hematopoiesis |
| wg | wingless | CG4889 | Hematopoiesis |
| wts | warts | CG12072 | Hematopoiesis |
| ytr | yantar | CG18426 | Hematopoiesis |
| zfh1 | Zn finger homeodomain 1 | CG1322 | Hematopoiesis |
| Cp19 | Chorion protein 19 | CG6524 | Melanization |
| Cyp4e3 | Cyp4e3 | CG4105 | Melanization |
| Cyp9f2 | Cyp9f2 | CG11466 | Melanization |
| Ddc | Dopa decarboxylase | CG10697 | Melanization |
| Hayan | Hayan | CG6361 | Melanization |
| Mco1 | Multicopper oxidase-1 | CG3759 | Melanization |
| MP1 | Melanization Protease 1 | CG1102 | Melanization |
| ple | pale | CG10118 | Melanization |
| PPO1 | Prophenoloxidase 1 | CG5779 | Melanization |
| PPO2 | Prophenoloxidase 2 | CG8193 | Melanization |
| PPO3 | Prophenoloxidase 3 | CG42640 | Melanization |
| Pu | Punch | CG9441 | Melanization |
| serpin-27A | Serpin 27A | CG11331 | Melanization |
| SP7 | Serine protease 7 | CG3066 | Melanization |
| yellow | yellow | CG3757 | Melanization |
| yellow-b | yellow-b | CG17914 | Melanization |
| yellow-c | yellow-c | CG4182 | Melanization |
| yellow-e | yellow-e | CG9792 | Melanization |
| Cht12 | Chitinase 12 | CG30293 | Wound_healing |
| Cht2 | Chitinase 2 | CG2054 | Wound_healing |
| Cht5 | Chitinase 5 | CG9307 | Wound_healing |
| Cht7 | Chitinase 7 | CG1869 | Wound_healing |
| Cht9 | Chitinase 9 | CG10531 | Wound_healing |
| Idgf1 | Imaginal disc growth factor 1 | CG4472 | Wound_healing |
| Idgf2 | Imaginal disc growth factor 2 | CG4475 | Wound_healing |
| Idgf3 | Imaginal disc growth factor 3 | CG4559 | Wound_healing |
| Idgf4 | Imaginal disc growth factor 4 | CG1780 | Wound_healing |

**Table S3. Scripts used for running LASSI Plus, exemplified for G. pusilla**

Short read alignment to species specific reference genome

#!/bin/bash

PATH=$PATH:/data/programs/bwa-mem2-2.2.1_x64-linux/

# rename reference to your reference and rootname to what you want your final bamfile to have as a suffix.

reference=gp_final.fa

suffix=gpus_aln

fq_list=gpus_fq

bwa-mem2 index refs/$reference

while read p

do

bwa-mem2 mem -t 40 refs/"$reference" fq/$p fq/${p%1.gz}2.gz | samtools view -Sb -F4 -@8 - -o bam/${p%1.gz}_v_$suffix.bam

done < $fq_list

# sort and index bamfiles

for sample in bam/*_v_$suffix.bam

do

samtools sort -@ 20 -o ${sample%bam}sorted.bam ${sample}

samtools index -@ 20 ${sample%bam}sorted.bam

done

-------------------------

Variant calling

#!/bin/bash

ls bam/G_pus*sorted.bam > gpus_bam

ref=gp_final.fa

cut -f1 refs/$ref.fai > gpus_chromo

cat gpus_chromo | \

parallel -j 40 "bcftools mpileup -Ou -f refs/$ref -b gpus_bam --annotate FORMAT/AD,FORMAT/ADF,FORMAT/ADR,FORMAT/DP,FORMAT/SP,INFO/AD,INFO/ADF,INFO/ADR -r {} | \

bcftools call -f GQ,GP -m -Ob -o gpus_vcf/Gpusilla_v_gp_raw_{}.bcf"

-------------------------

Variant filtering

#mkdir gpus_bcf_noindel

#mkdir gpus_vcf_filt

#cd gpus_vcf

#ls *bcf > ../gpus_raw_bcf

#cd ../

ref=gp_final.fa

# filters for popgen stats

# remove indels

#ls ls cat gpus_raw_bcf | parallel -j 20 "bcftools view --exclude-types indels -O u -o gpus_bcf_noindel/{}.noIndel.bcf gpus_vcf/{}"

# filter based on quality and depth

cd gpus_bcf_noindel

for i in *bcf

do bcftools view -i 'QUAL>30 & AVG(FMT/DP)>5 & AVG(FMT/DP)<100' -O z -o ../gpus_vcf_filt/${i%.bcf}.q20.dp5_100.vcf.gz $i

done

cd ../gpus_vcf_filt

ls gpus_vcf_filt/*vcf.gz > gpus_filt_vcf

bcftools concat -n --threads 50 -Oz -o gpus_v_gp_bcftools_noIndel_q20_dp5_100_all.vcf.gz -f gpus_filt_vcf

tabix gpus_v_gp_bcftools_noIndel_q20_dp5_100_all.vcf.gz --threads 20

#bcftools concat -n --threads 20 -Ob -o 44_sample_v_gc_bcftools_raw_all.bcf -f raw_bcf

#tabix 44_sample_v_gc_bcftools_raw_all.bcf

#bcftools view --max-alleles 2 vcf/44_sample_v_gc_bcftools_noIndel_q20_dp5_100_all.vcf.gz -Oz -o vcf/44_sample_v_gc_bcftools_noIndel_q20_dp5_100_all_MM2.vcf.gz --threads 20

-------------------------

Running LASSI Plus

#!/bin/bash

#SBATCH -A naiss2023-22-26

#SBATCH -p node

#SBATCH -t 3-00:00:00

#SBATCH -J 44sampls_v_gp_lassi-gpus_51_12

#SBATCH -D /proj/uppstore2017082/kalle_analysis/

#SBATCH --mail-user=kalle.tunstrom@zoologi.su.se

#SBATCH --mail-type=ALL

module load bioinfo-tools

module load bcftools htslib samtools

lassi=/proj/uppstore2017082/kalle_analysis/salti/lassip/src

pop=/proj/uppstore2017082/kalle_analysis/gpus_pop

#vcf=/proj/uppstore2017082/kalle_analysis/vcf/44_sample_v_gc_bcftools_noIndel_q20_dp5_100_all_MM2.vcf.gz

#$lassi/lassip --vcf $vcf --hapstats --pusc-spec --lassi --pop $pop --out ${vcf%vcf.gz} --winsize 102 --winstep 51 --unphased --threads 20

#done

#$lassi/lassip --spectra lassi/*._51_12.gpus.lassip.mlg.spectra.gz --lassi --out gpus_long100kb_51_12.LASSI --threads 20

#echo HEY

$lassi/lassip --spectra gpus_lassi/*.spectra.gz --salti --out gpus_v_gp_51_12.SALTI --threads 20

**Table S4.** Selection intensities (S.I.), p-values and θ from HDMKPRF for included candidate immune genes (green = positive S.I., red = negative S.I.). Observed gene expression (DEX) in *G. pusilla* from an infection experiment with *A. parviclava* are included (DE+ = upregulated, DE- = downregulated, NA = data insufficient, NI = not analysed). In *G. calmariensis*, only one gene (stam) was differentially expressed but this gene could not be analyzed here. The third species (*G. tenella*) was not included in the gene expression analysis.

|  |  | *G. pusilla* | | | *G. tenella* | | *G. calmariensis* | |  |  |
| --- | --- | --- | --- | --- | --- | --- | --- | --- | --- | --- |
| Gene | Function | DEX | S.I. | p | S.I. | p | S.I. | p | θr | θs |
| alphaPS4 | Recognition | DE- | -0.79 | 0.0188 | -1.74 | 0.0001 | -1.47 | 0.0003 | 20.75 | 1.87 |
| Corin | Recognition | NA | 1.94 | 0.9980 | 1.94 | 0.9980 | 0.87 | 0.9081 | 4.07 | 5.21 |
| GNBP3 | Recognition | DE+ | -2.05 | 0.3970 | -1.63 | 0.4070 | -1.82 | 0.4116 | 0.00 | 0.08 |
| Hml | Recognition | 0 | -1.55 | 0.0000 | -1.72 | 0.0000 | -1.70 | 0.0000 | 41.65 | 9.69 |
| lectin-24A | Recognition | NA | -3.67 | 0.0750 | -5.57 | 0.0293 | 1.41 | 0.8316 | 1.98 | 0.98 |
| PGRP-LB | Recognition | 0 | -5.64 | 0.0309 | -6.22 | 0.0233 | -8.76 | 0.0074 | 3.27 | 1.30 |
| PGRP-LE | Recognition | 0 | 4.63 | 0.9548 | 5.81 | 0.9837 | 4.88 | 0.9578 | 0.24 | 2.36 |
| PGRP-SA | Recognition | DE+ | -0.21 | 0.3916 | -8.36 | 0.0041 | -6.61 | 0.0122 | 2.79 | 0.65 |
| PGRP-SB1 | Recognition | 0 | -1.78 | 0.2060 | -4.19 | 0.0735 | 0.47 | 0.5589 | 1.98 | 1.47 |
| PGRP-SD | Recognition | 0 | -6.57 | 0.0129 | -5.17 | 0.0265 | -9.83 | 0.0026 | 4.02 | 3.01 |
| santa-maria | Recognition | 0 | 6.25 | 0.9995 | 3.45 | 0.9633 | 1.45 | 0.7141 | 0.58 | 2.20 |
| Tep4 | Recognition | NI | 0.10 | 0.5590 | 0.13 | 0.5728 | 0.63 | 0.8691 | 6.73 | 2.04 |
| CG2736 | Recognition | DE+ | -7.51 | 0.0116 | -7.90 | 0.0090 | -4.88 | 0.0377 | 3.57 | 1.79 |
| 18w | Signaling | 0 | -2.43 | 0.0453 | -4.72 | 0.0097 | -1.96 | 0.0331 | 4.98 | 11.32 |
| bsk | Signaling | 0 | -4.86 | 0.2301 | -0.36 | 0.4856 | -4.75 | 0.2462 | 0.15 | 2.44 |
| dl | Signaling | 0 | 0.38 | 0.5866 | -1.38 | 0.1300 | -2.24 | 0.1251 | 2.24 | 3.01 |
| DmMyD88 | Signaling | 0 | 3.76 | 0.9105 | -2.63 | 0.2699 | -0.35 | 0.4732 | 0.58 | 0.98 |
| dome | Signaling | 0 | 0.09 | 0.5394 | -1.19 | 0.0324 | -3.99 | 0.0002 | 8.23 | 1.06 |
| grass | Signaling | DE+ | 3.07 | 0.9814 | 0.97 | 0.7210 | 2.94 | 0.9744 | 1.05 | 4.56 |
| hep | Signaling | 0 | -6.63 | 0.1288 | -0.18 | 0.4923 | -6.81 | 0.1361 | 0.36 | 1.14 |
| IKKβ | Signaling | 0 | -1.14 | 0.1683 | -0.68 | 0.2130 | -0.49 | 0.2872 | 3.48 | 2.93 |
| imd | Signaling | 0 | 0.12 | 0.5271 | -8.23 | 0.0029 | -2.20 | 0.0330 | 2.65 | 0.73 |
| Jra | Signaling | 0 | -2.19 | 0.3857 | -1.73 | 0.4063 | -1.91 | 0.4085 | 0.00 | 1.79 |
| Kenny | Signaling | 0 | 1.00 | 0.7918 | -0.31 | 0.4034 | 0.17 | 0.5149 | 2.23 | 2.77 |
| pll | Signaling | 0 | -5.09 | 0.0013 | -2.42 | 0.0205 | -2.46 | 0.0051 | 7.37 | 5.05 |
| psh | Signaling | NI | 0.17 | 0.4572 | -4.15 | 0.1188 | -3.70 | 0.1424 | 1.46 | 2.04 |
| Rel | Signaling | DE+ | -0.26 | 0.3127 | 1.02 | 0.9581 | 0.19 | 0.6087 | 5.83 | 0.82 |
| SPE | Signaling | DE+ | -4.26 | 0.2040 | -4.24 | 0.1908 | 3.30 | 0.8428 | 0.47 | 2.28 |
| spirit | Signaling | NI | -6.50 | 0.0926 | -4.27 | 0.1669 | -6.62 | 0.0909 | 1.07 | 1.96 |
| Stat92E | Signaling | 0 | -2.10 | 0.0007 | -2.01 | 0.0021 | -2.91 | 0.0000 | 13.84 | 0.49 |
| Tak1 | Signaling | 0 | 6.09 | 0.9979 | -0.02 | 0.5107 | 1.52 | 0.7084 | 0.59 | 4.15 |
| Toll-6 | Signaling | 0 | -7.14 | 0.0146 | -8.94 | 0.0053 | -3.60 | 0.0713 | 3.33 | 10.01 |
| Toll-7 | Signaling | 0 | -3.41 | 0.0887 | 1.19 | 0.7953 | -0.61 | 0.2917 | 1.82 | 10.67 |
| Tollo | Signaling | DE- | 0.21 | 0.5369 | 0.38 | 0.5969 | 0.21 | 0.5248 | 2.03 | 12.05 |
| Cyp6a17 | Effector | 0 | -1.77 | 0.0294 | -3.73 | 0.0071 | -7.41 | 0.0003 | 7.06 | 5.05 |
| Cyp12a4 | Effector | DE+ | -0.73 | 0.2118 | -8.93 | 0.0001 | -2.54 | 0.0064 | 5.39 | 3.18 |
| Cyp12e1 | Effector | NA | -2.80 | 0.3125 | 5.75 | 0.9707 | 0.55 | 0.5637 | 0.27 | 0.81 |
| DHPR | Effector | 0 | 4.44 | 0.9284 | 0.10 | 0.5287 | -1.21 | 0.4061 | 0.44 | 0.73 |
| Spn28D | Protease | DE+ | -9.26 | 0.0239 | -6.38 | 0.0750 | -0.94 | 0.3855 | 1.07 | 3.51 |
| spn4 | Protease | NA | -1.99 | 0.1091 | -1.73 | 0.0732 | -3.04 | 0.0563 | 3.44 | 3.74 |
| Spn88Eb | Protease | 0 | -4.74 | 0.2381 | -4.15 | 0.2489 | 0.52 | 0.5419 | 0.14 | 0.65 |
| Tequila | Protease | 0 | -0.59 | 0.0666 | -0.56 | 0.0711 | -0.49 | 0.0995 | 16.19 | 2.12 |
| λTry | Protease | 0 | -0.13 | 0.4312 | -4.73 | 0.0058 | -0.42 | 0.2685 | 4.26 | 2.52 |
| CG30371 | Protease | DE- | -3.02 | 0.0021 | -1.42 | 0.0962 | -3.29 | 0.0111 | 5.65 | 2.77 |
| CG3117 | Protease | 0 | -2.97 | 0.2997 | -3.05 | 0.2835 | 4.22 | 0.8773 | 0.32 | 3.26 |
| CG31827 | Protease | 0 | -9.14 | 0.0042 | -5.65 | 0.0272 | -6.18 | 0.0205 | 3.64 | 1.96 |
| CG32374 | Protease | 0 | 2.36 | 0.7681 | -5.06 | 0.1383 | -2.28 | 0.3080 | 0.62 | 1.63 |
| CG32483 | Protease | NI | 4.78 | 0.9886 | 1.22 | 0.6736 | 1.07 | 0.6775 | 0.62 | 1.22 |
| CG3344 | Protease | 0 | -5.17 | 0.0016 | -9.91 | 0.0000 | -5.67 | 0.0007 | 10.38 | 2.12 |
| CG3916 | Protease | 0 | -3.64 | 0.1910 | 1.51 | 0.6928 | -6.30 | 0.0852 | 0.85 | 1.31 |
| CG4053 | Protease | 0 | -8.88 | 0.0349 | -2.10 | 0.3023 | -6.15 | 0.1123 | 0.94 | 3.34 |
| CG6041 | Protease | 0 | -3.37 | 0.1464 | -8.34 | 0.0187 | -6.60 | 0.0515 | 1.96 | 2.20 |
| CG6639 | Protease | 0 | -5.33 | 0.2049 | 5.87 | 0.9559 | -5.24 | 0.2137 | 0.14 | 3.09 |
| CG9240 | Protease | 0 | -1.92 | 0.3478 | -4.55 | 0.1849 | -8.14 | 0.0703 | 0.64 | 0.33 |
| brahma | Hematopoiesis | 0 | -3.22 | 0.1425 | -2.67 | 0.1758 | -0.65 | 0.2886 | 1.80 | 6.51 |
| cher | Hematopoiesis | 0 | 2.00 | 0.9892 | 9.43 | 1.0000 | 0.56 | 0.7529 | 2.70 | 0.74 |
| Cyt-b5 | Hematopoiesis | DE+ | -2.59 | 0.2668 | 7.33 | 0.9999 | 1.24 | 0.6446 | 0.54 | 0.81 |
| Dronc | Hematopoiesis | 0 | 0.70 | 0.6230 | 0.90 | 0.7018 | 0.78 | 0.6142 | 1.18 | 2.04 |
| Dsor1 | Hematopoiesis | 0 | -6.06 | 0.1106 | -2.15 | 0.3028 | -9.18 | 0.0374 | 0.95 | 2.36 |
| Egfr | Hematopoiesis | 0 | -8.03 | 0.0130 | -6.89 | 0.0229 | -4.97 | 0.0515 | 2.85 | 11.72 |
| gcm | Hematopoiesis | NA | -10.44 | 0.0019 | -2.72 | 0.1098 | -8.79 | 0.0058 | 3.21 | 3.18 |
| kn | Hematopoiesis | NA | -2.33 | 0.3807 | -1.68 | 0.4064 | -1.77 | 0.4127 | 0.00 | 2.61 |
| lwr | Hematopoiesis | 0 | -6.62 | 0.1294 | -0.17 | 0.4955 | -6.98 | 0.1308 | 0.36 | 1.79 |
| lz | Hematopoiesis | 0 | -5.20 | 0.1140 | -7.27 | 0.0502 | -5.20 | 0.1166 | 1.39 | 3.75 |
| Notch | Hematopoiesis | 0 | -1.13 | 0.3767 | 3.48 | 0.9590 | 3.24 | 0.9307 | 0.65 | 12.95 |
| pnt | Hematopoiesis | DE+ | 1.12 | 0.5757 | -0.07 | 0.4960 | -2.75 | 0.2743 | 0.70 | 1.87 |
| Pvf2 | Hematopoiesis | DE+ | 0.47 | 0.5484 | -0.67 | 0.3670 | -4.50 | 0.0761 | 1.71 | 1.55 |
| Pvr | Hematopoiesis | 0 | -4.80 | 0.0000 | -9.18 | 0.0000 | -6.91 | 0.0000 | 48.92 | 3.99 |
| Rac1 | Hematopoiesis | 0 | -2.23 | 0.3854 | -1.55 | 0.4127 | -1.99 | 0.4013 | 0.00 | 1.30 |
| raf | Hematopoiesis | 0 | -0.60 | 0.4653 | 2.77 | 0.7699 | -0.39 | 0.4791 | 0.19 | 0.46 |
| Ser | Hematopoiesis | 0 | -5.13 | 0.0743 | -5.51 | 0.0635 | -9.15 | 0.0157 | 2.13 | 5.21 |
| srp | Hematopoiesis | DE+ | -10.67 | 0.0053 | -2.68 | 0.1901 | -6.53 | 0.0537 | 1.88 | 1.79 |
| Su(H) | Hematopoiesis | 0 | -6.68 | 0.1288 | -0.26 | 0.4911 | -6.89 | 0.1264 | 0.36 | 4.15 |
| ush | Hematopoiesis | 0 | -5.97 | 0.0419 | -6.42 | 0.0345 | -7.73 | 0.0206 | 2.57 | 4.48 |
| wg | Hematopoiesis | 0 | -2.03 | 0.3969 | -1.70 | 0.4049 | -1.53 | 0.4270 | 0.00 | 1.06 |
| wts | Hematopoiesis | DE+ | -5.36 | 0.0749 | 0.75 | 0.6094 | -7.70 | 0.0293 | 1.31 | 9.77 |
| yantar | Hematopoiesis | 0 | -0.02 | 0.5110 | -4.09 | 0.2504 | -4.72 | 0.2492 | 0.15 | 0.57 |
| zfh1 | Hematopoiesis | DE+ | 2.92 | 0.9801 | -0.03 | 0.4542 | 0.97 | 0.7655 | 1.44 | 1.63 |
| Cyp4e3 | Melanization | DE+ | 0.84 | 0.6539 | -0.49 | 0.3281 | -7.36 | 0.0382 | 1.12 | 2.44 |
| Cyp9f2 | Melanization | DE+ | -2.57 | 0.2599 | 1.50 | 0.7225 | 9.78 | 1.0000 | 0.55 | 4.64 |
| Ddc | Melanization | DE+ | -9.38 | 0.0022 | -8.30 | 0.0045 | -2.79 | 0.0799 | 3.84 | 4.64 |
| Hayan | Melanization | 0 | -5.20 | 0.0712 | -7.14 | 0.0311 | -6.99 | 0.0366 | 2.15 | 1.95 |
| Mco1 | Melanization | 0 | -7.32 | 0.0017 | -5.18 | 0.0072 | -9.70 | 0.0003 | 7.06 | 5.13 |
| MP1 | Melanization | NI | -2.93 | 0.3076 | -2.90 | 0.3017 | -6.88 | 0.1323 | 0.39 | 1.96 |
| ple | Melanization | 0 | -3.88 | 0.1950 | -6.24 | 0.0942 | -6.53 | 0.1007 | 1.04 | 3.74 |
| PPO2 | Melanization | DE- | -8.34 | 0.0042 | -7.62 | 0.0069 | -4.74 | 0.0252 | 4.32 | 5.05 |
| Pu | Melanization | DE+ | -2.90 | 0.3103 | -3.05 | 0.2946 | -7.02 | 0.1252 | 0.40 | 0.33 |
| serpin-27A | Melanization | 0 | -5.00 | 0.2248 | -0.31 | 0.4916 | -4.86 | 0.2435 | 0.15 | 1.47 |
| SP7 | Melanization | DE+ | -4.85 | 0.2322 | -4.03 | 0.2573 | 0.20 | 0.5325 | 0.14 | 2.77 |
| yellow | Melanization | DE+ | -11.23 | 0.0004 | -2.24 | 0.1041 | -9.82 | 0.0013 | 4.03 | 3.50 |
| yellow-c | Melanization | DE- | 2.57 | 0.9647 | 1.52 | 0.8269 | 2.08 | 0.9107 | 1.06 | 2.77 |
| Cht2 | Wound healing | DE+ | -2.41 | 0.1994 | -10.14 | 0.0057 | -4.89 | 0.0892 | 2.01 | 3.34 |
| Cht5 | Wound healing | DE+ | -0.98 | 0.1037 | -0.32 | 0.3151 | -0.75 | 0.1461 | 5.28 | 0.81 |
| Cht7 | Wound healing | 0 | 0.54 | 0.8214 | 0.98 | 0.9535 | 0.30 | 0.6856 | 5.84 | 0.73 |
| Cht9 | Wound healing | 0 | -8.60 | 0.0005 | -0.04 | 0.4573 | -10.30 | 0.0001 | 4.36 | 3.26 |
| Idgf4 | Wound healing | 0 | 2.39 | 0.7828 | -0.86 | 0.4213 | -8.49 | 0.0470 | 0.59 | 2.77 |
